# DOSE: An open-source, iOS watch-based tool for experience sampling

**DOI:** 10.1371/journal.pdig.0001627

**Published:** 2026-08-17

**Authors:** Ian Kim, Sahiti Kunchay, Saeed Abdullah, David E. Conroy

**Affiliations:** 1 Department of Kinesiology, Penn State University, University Park, Pennsylvania, United States of America; 2 Department of Psychology, Stanford University, Stanford, California, United States of America; 3 Department of Pediatrics, Stanford University, Stanford, California, United States of America; 4 Department of Psychiatry, Yale University, New Haven, Connecticut, United States of America; 5 College of Information Sciences and Technology, Penn State University, University Park, Pennsylvania, United States of America; 6 School of Kinesiology, University of Michigan, Ann Arbor, Michigan, United States of America; Tehran University of Medical Sciences, IRAN, ISLAMIC REPUBLIC OF

## Abstract

Smartwatches facilitate low-burden rapid-access micro-interactions, making them ideal for Experience Sampling Methods (ESMs). Despite the Apple Watch being the most popular smartwatch in the U.S., its broad use in ESM studies has been limited by a lack of accessible frameworks that enable deployment without technical expertise. We developed DOSE, an open-source ESM framework tailored for the Apple Watch. It includes tools and documentation that allow researchers to configure surveys, build custom apps, deploy studies, and stream data to servers with minimal familiarity with Xcode and iOS development workflows. We evaluated the framework’s feasibility in a 28-day field study with 18 participants (mean age = 55.3 ± 9.2). Results showed reliable prompt delivery; middle-aged and older adults achieved median survey completion rates of 69% and 88% during watch wear periods, median device access times of 9 and 9.5 seconds, and median total response times of 22 and 29 seconds, respectively. Participants generally demonstrated increasing efficiency in responses over time. These findings establish the DOSE framework as a practical, feasible solution for Apple Watch-based ESMs and a foundation for future smartwatch research.

## Author summary

Smartwatches offer a convenient way for people to share real-time information about their experiences, habits, and behaviors throughout the day. Despite the Apple Watch being the most widely used smartwatch in the U.S., it has not been widely used in research studies that rely on frequent, short surveys, largely because there has not been an easy-to-use system for researchers without programming experience. To address this, we developed DOSE, an open-source framework that lets researchers design surveys, create custom apps, run studies, and securely collect data from participants’ Apple Watches, with minimal familiarity with Xcode and iOS development workflows. We tested DOSE in a 28-day study with 18 adults and found that prompts were delivered reliably, responses were quick (often under 10 seconds), and participants became more efficient over time. This demonstrates that DOSE makes high-frequency, real-world data collection on the Apple Watch practical and scalable, laying the groundwork for future research in clinical studies, behavior tracking, and personalized health.

## Introduction

The Experience Sampling Method (ESM) is commonly employed to study individuals in their natural environments. ESM studies gather responses from repeated assessments at moments over time, from minutes to days to months, with the intent of obtaining lived, day-to-day experience of individuals, while minimizing retrospective biases that plague single-timepoint, between-person assessments [1–4]. The technology on which this research method to solicit respondents’ answers at random moments of the day is based has evolved extensively, moving from paper diaries to pagers to personal digital assistants to connected mobile devices [5, 6]. However, the core features of the method have remained essentially unchanged since its inception in the 1970s and its future evolution will likely be driven by technological advances that support remote data collection, monitoring, and intervention. Wearable devices, such as smartwatches, represent one of the latest technological advances for ESM; however, existing applications have concentrated on Android, Fitbit, and maker applications because of constraints in Apple iOS. In this paper, we provide the code for Apple Watch-based experience sampling that overcomes a limitation in the Apple SDK to permit extended monitoring periods.

Smartwatches offer several advantages over smartphones for this type of work. First and foremost, smartwatches are worn on the wrist, increasing proximity and potentially reducing response latencies. Increased proximity can improve sampling coverage by reducing the likelihood of systematically missing responses due to the smartphone not being accessible (e.g., while exercising, when the smartphone is stored in a desk drawer or purse). Improved coverage will yield more valid estimates of the momentary experiences and their dynamics. Previous studies have demonstrated higher compliance rates when using smartwatches for ESM (65% on smartphone vs. 76% on smartwatch), indicating improved participant engagement and adherence to data collection protocols [7–11]. Smartwatches also have fewer distractions compared to smartphones (i.e., receiving fewer notifications and providing less access to social media apps), allowing participants to maintain focus on ESM prompts and responses. An additional advantage that distinguishes smartwatches from smartphones on ESM is their support for micro-interactions [9], defined as brief interactions in which a user retrieves a device and completes a single-item questionnaire, with each phase being completed within seconds. This can significantly reduce participant burden and enable more intensive sampling over time [9]. Additionally, smartwatches have attracted attention for their ability to readily link and align self-report data with markers from other sensors within the watch, such as HealthKit data. This integration allows for a comprehensive and holistic understanding of the user’s health and well-being.

More than half of adults in the US own a smartwatch [12, 13]. However, the adoption of smartwatches generally and their use for ESM specifically are still at an early stage. The limited studies using smartwatches for ESM have largely focused on evaluating the feasibility of the approach. The few existing smartwatch-based ESM apps are typically only available on the Android platform or custom hardware [14–18]. None are available for the Apple Watch despite its dominant position in the smartwatch industry, boasting over 115 million active users and a 59% market share in the US [19, 20]. This discrepancy has resulted in systematic exclusion of a large segment of the population from smartwatch-based ESM research.

The primary reason for the Android platform’s dominance in ESM studies is that, as of June 2026, there are some key features that are harder to implement on iOS-based smartwatch ESM apps compared to Android-based smartwatch apps. First, iOS imposes restrictions on the number of concurrently scheduled local notifications per app. Specifically, the system maintains a maximum of 64 pending notifications closest to their scheduled prompting time; any notifications exceeding this limit are discarded by the system. Assuming a frequency of six daily surveys, ESM studies would exhaust 42 notification slots within a single week, implying that a fully pre-scheduled design would reach the 64-notification limit in approximately two weeks. In contrast, Android does not impose the same constraint, allowing greater flexibility for long-horizon prompting designs.

Second, iOS does not provide a built-in mechanism specifically design for automatic randomized scheduling of notifications in ESM studies. In contrast, Android provides various APIs and features, including *AlarmManager, JobScheduler* and *WorkManager*, that support flexible scheduling of tasks and can more readily accommodate programmatically generated or randomized prompting schedules. Manually assigning random schedules within the iOS app code is generally not recommended due to privacy risks, limited adoptability (i.e., ability to modify schedules dynamically during a study to accommodate changes in participant availability, preferences, or study requirements) and limited scalability (i.e., difficulty in managing a large number of participants). Releasing an iOS app with participant IDs via the App Store or TestFlight also requires additional steps due to data protection and ethical concerns, even when IDs were randomly generated by a computer [21]. Implementing a remote configuration system where the schedules are stored on a server or content management system can be an alternative. However, this approach adds complexity to the app architecture, requiring extra networking code and error handing for retrieval and synchronization of schedule data. Moreover, its reliance on external servers introduces potential failure points, such as missed schedules due to server downtime or connectivity problems.

Lastly, the closed-source nature of iOS has limited the extent of active discussions and comprehensive documentation surrounding app development. In contrast to open-source platforms like Android, where developers actively collaborate, the iOS ecosystem offers fewer opportunities for community-driven discussions and knowledge sharing. Developing a new app from the scratch requires a significant investment of time and resources for researchers. The development process typically involves conceptualization, design, coding, testing, and deployment, which can span several months or longer. Financial investment is also often necessary, particularly for hiring skilled developers and app testers, posing significant challenges for projects with limited budgets. Although existing and well-tested ESM app platforms can help reduce development time and costs by enabling researchers to adapt prebuilt solutions to their specific needs, no publicly available or customizable platform currently exists for iOS smartwatch-based ESM research app development.

## Results

**Fig 1:**
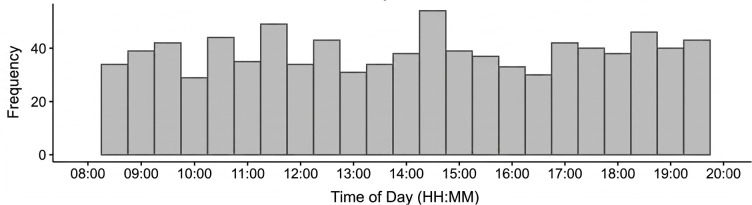
Distribution of notification prompt deliveries.

The study app, built on the DOSE framework, successfully delivered randomized survey prompts three times daily within predefined 4-hour time windows over the 28-day study period, totaling 84 notifications—surpassing the standard iOS scheduling limit of 64 (Fig 1). Surveys were delivered without technical interruptions or scheduling failures, demonstrating both the feasibility of our extended notification scheduling approach and the practicality of using DOSE framework for experience sampling.

**Fig 2:**
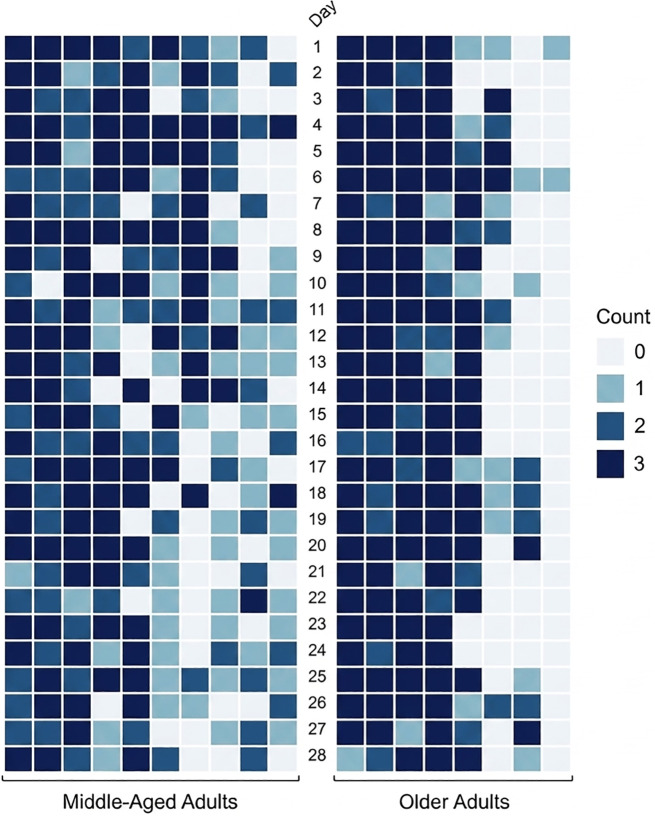
Heatmaps showing the number of daily completed surveys by participants over 28 study days. The vertical axis represents study days (1 to 28), while each column corresponds to an individual participant. Color intensity indicates the survey completion frequency, with darker shades representing higher counts.

On valid days—when participants wore their Apple Watch—survey completion rates averaged 70% (median 69%) for middle-aged adults and 74% (median 88%) for older adults (Table 1). Despite higher completion rates, older adults showed a lower mean proportion of valid windows than middle-aged adults (but this finding appeared to be driven by three participants who struggled with watch wear, as reflected in the discrepancy between mean and median values). Among incomplete surveys, the proportions that were opened but not submitted—possibly due to interruptions, technical difficulties, or accidental dismissal (e.g., clumsy hands)—was 16% for middle-aged adults and 27% for older adults. Most middle-aged participants showed relatively consistent behavior, with occasional missed surveys or device non-wear; older adults, however, showed more variability: some rarely wore the watch, while others wore it consistently and completed most surveys (Fig 2).

**Table 1:** Device wear proportions and survey completion rates over the 28-day study period by age group.

	Valid Window Proportion (Watch Device Worn)		Completion Rate of Valid Windows (%)		Completion Rate of Intention-to-Prompt (%)	
Group	Mean (SD)	Median (IQR)	Mean (SD)	Median (IQR)	Mean (SD)	Median (IQR)
Middle-Aged Adults	77.38 (13.52)	72.62 (15.77)	70.06 (18.39)	69.17 (29.87)	55.12 (19.74)	55.36 (25.30)
Older Adults	70.09 (32.60)	82.74 (53.87)	74.42 (33.29)	87.89 (19.75)	54.46 (37.46)	61.31 (63.10)

Note: Proportions are aggregated at the participant level. *Valid Window Proportion* represents the percentage of total 4-hour blocks containing ≥ 1 hour of HealthKit heart rate data. *Completion Rate of Valid Windows* uses these wear-verified periods as the denominator. *Completion Rate of Intention-to-Prompt* evaluates compliance against the uncorrected absolute maximum theoretical baseline of 84 planned prompts; this baseline represents an upper boundary that assumes continuous device wear and regular active app openings, as background system updates are required to sustain consecutive prompt scheduling via iOS.

**Fig 3:**
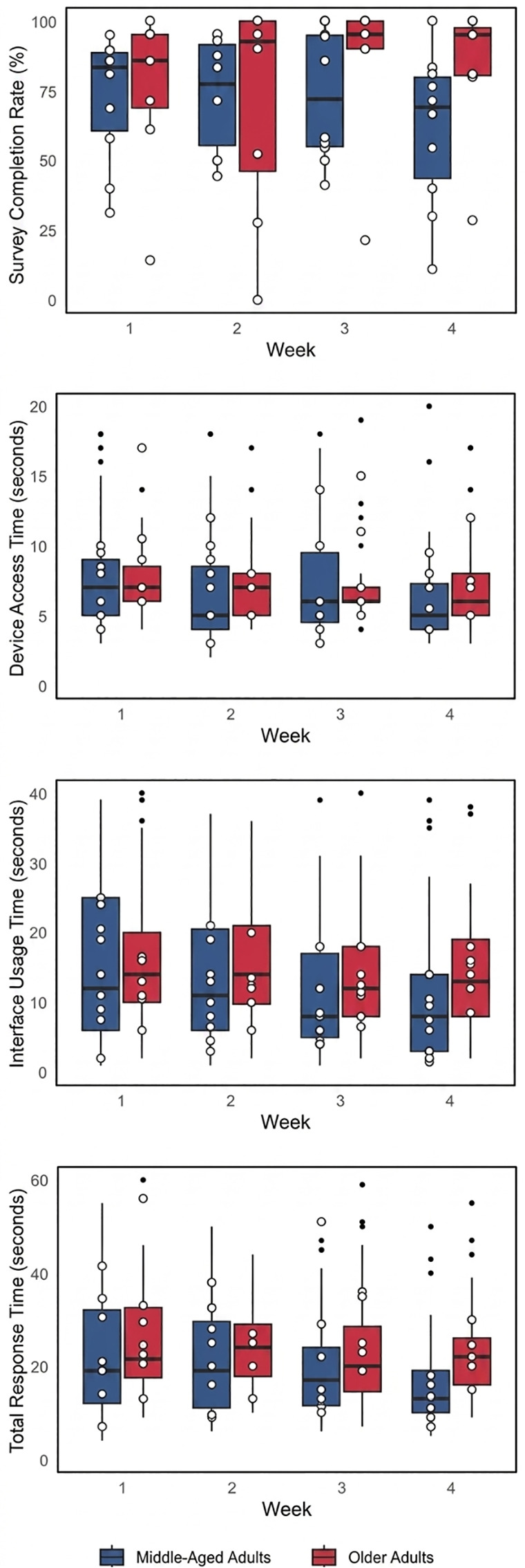
Weekly completion rates and interaction metrics by age group. For all panels, boxplots display the distribution interquartile range (IQR; box hinges), group median values (horizontal bar), and 1.5x IQR thresholds (whiskers). Black points located outside the whiskers indicate outliers. Overlaid white circles represent the average weekly performance metric for each unique participant.

Fig 3 shows the weekly completion rates, and distributions of time spent on device access, interface usage, and total response by age group. Over the four-week period, middle-aged adults showed a general downward trend in median survey completion rates, whereas older adults experienced an overall upward trend. The median device access time was 9 seconds (IQR = 32.2) for middle-aged adults and 9.5 seconds (IQR = 1241.0) for older adults (Table 2). Active survey completion within the application was swift and highly consistent across both groups; the median interface usage time was 10 seconds (IQR = 14) for middle-aged adults, with a micro-interaction rate of 24.1% (SD = 19.0%), and 13 seconds (IQR = 11) for older adults, with a micro-interaction rate of 6.9% (SD = 6.5%). The median total response time was 22 seconds (IQR = 39.2) for middle-aged adults and 29 seconds (IQR = 1256) for older adults. Notably, the pronounced magnitude of the IQRs observed for device access—particularly among older adults—reflects an elongated upper tail, where a distinct portion of prompts (24.7% for middle-aged and 34.5% for older adults) exceeded an access latency of 1 minute before completion, contributing to a matching skew in total survey completion times. For both middle-aged and older adults, all three time metrics—device access time, interface usage time, and total response time—consistently decreased across the weeks, suggesting that participants became more efficient with the technology and survey procedures through repeated use. Although we did not statistically compare these weekly trends between groups due to limited power from the small sample size, visual inspection suggests that, within this feasibility sample, middle-aged adults may have adapted more quickly than older adults. However, this descriptive pattern should be interpreted cautiously, as it may reflect the behavior of a small number of participants rather than a generalizable age-group characteristics.

**Table 2:** Distributional percentiles for survey interaction latencies and threshold proportions by age group.

		Interaction Latencies (in seconds)				Threshold Proportions (%)			
Group	Metric	25^th^	50^th^	75^th^	90^th^	95^th^	>1 min	>3 min	>5 min
**Middle-Aged Adults**	Device Access	5	9	37.2	2,454	4,419	24.7	22.5	21.8
	Interface Usage	5	10	19	27	34	0	0	0
	Total Response	12.8	22	52	2,476.5	4,431.8	24.7	22.5	21.8
**Older Adults**	Device Access	6	9.5	1,247	3,225.1	4,506.6	34.5	32.8	32.1
	Interface Usage	9	13	20	27	32	0	0	0
	Total Response	20	29	1,276	3,240.1	4,525	34.5	32.8	32.1

## Discussion

This paper introduced DOSE, a new app for research using ESM on Apple Watches and reported on the feasibility of deploying the app with middle-aged and older adults. The wearable nature of smartwatches makes them an intriguing method platform for ESM but idiosyncrasies in the Watch iOS have limited applications of the Apple Watch for experience sampling schedules with more than 64 randomly-scheduled prompts. The new DOSE app for Watch OS circumvents that limitation and enables researchers to deliver randomly-scheduled prompts without an upper limit on study duration. Feasibility testing revealed survey completion rates above 75% for both middle-aged and older adults. However, interpretations of age-group differences should be made cautiously because the feasibility study included only eight older adults, and several observed patterns may reflect individual-level variability rather than generalizable age-related characteristics. Some older adults appeared to not wear their watches on certain days, suggesting that device wear may be an important consideration when developing sampling protocols for older adults. Responses were relatively quick following prompts for both middle-aged and older adults; however, older adults had some prolonged response times so future deployments should verifying that users activate notification permissions from their Watch. Participant generally completed responses within 30 seconds of receiving a prompt. Over the four-week test period, participants in both age groups appeared to become more proficient with the app as evidenced by faster response times. This work makes three significant contributions to the literature.

First, the DOSE app facilitates broader implementation of ESM via smartwatches by successfully circumventing a limitation of the Apple Watch OS that precluded extended sampling schemes. Smartphones are currently the dominant technology for conducting research with ESM. The first smartwatches were introduced to consumers just over a decade ago and have since emerged as an industry with over $10B sales in the US and over $35B (Fortune Business [22]. Smartwatches offer one key advantage over smartphones – they are designed to be worn on the person which increases proximity for responding to notifications. One barrier to using smartwatches for ESM data collection is the limited number of suitable apps. For Android devices, researchers have developed several options tailored to their needs [9, 15, 23]. The fragmented nature of the Android smartwatch ecosystem—where apps developed for one device are often incompatible with others—have often led researchers to focus on one or a few specific Android smartwatch models or non-commercial devices [9, 15, 18, 24, 25]. For Fitbit smartwatches, Fitabase developed a platform called Engage to enable ecological momentary assessments (including ESM) [26]; however, we are not aware of any open-source solutions for those devices. The DOSE app provides a new and, to the best of our knowledge, the first, open-source option for conducting ESM research on the Apple Watch over long time intervals. The DOSE framework specifically addresses these challenges through custom functions for dynamic notification scheduling and random date/time selection, enabling researchers to configure study duration, time windows for notifications, and prompt frequency via user-defined parameters tailored to specific research needs. As the first open-source framework for developing ESM applications on Apple Watch, DOSE not only expands platform accessibility but also enhances the inclusivity, flexibility, and long-term applicability of ESM research.

Prior research on smartwatch use for experience sampling has largely focused on young and middle-aged adults. Developing technologies for older adults will be essential for facilitating their representation in research. A major demographic transition is underway in the US and the Census Bureau projects that the country will have more older adults than children or youth within a decade [27]. Despite many negative stereotypes about aging adults’ technology use, mobile technology adoption continues to increase among middle-aged and older adults [28, 29]. Adoption of wearable technologies, such as smartwatches, lags behind somewhat but will inevitably catch up as existing older adults adopt new technologies and younger cohorts who have adopted those technologies age in [16, 30, 31].

Results from our feasibility study add to accumulating evidence that older adults can use these devices. For example, one recent study explored the potential of smartwatches for ESM in older adults, finding that participants generally viewed smartwatch technology positively and expressed interest in its real-time assessment capabilities, but concerns about small screens and interface complexity were noted [16]. Similarly, another study reported that while general perceptions of seniors’ use of smartwatches often focus on cognitive aging, older adults themselves identified the difficulty of reading and interpreting smartwatch displays as a key barrier to adoption [30]. While two recent studies explored the use of smartwatches with older adults, the devices were primarily used as activity trackers in lab-based setups, with no experience sampling conducted [24, 32]. To our knowledge, our study is among the first to employ smartwatches for experience sampling in middle-aged and older adults, establishing the real-world feasibility of smartwatch-based ESM studies in this population. Our study demonstrated that middle-aged and older adults are both willing and able to engage with an Apple Watch app for self-monitoring. The greatest challenge for future work with older adults involves promoting daily device wear; however, we caution that these observations were based on a small sample and may have been disproportionately influenced by a small number of individuals rather than a generalizable characteristic of older adults. Slight increases in response times compared to prior work likely resulted from the naturalistic design of our study [9]. Unlike prior research with strict constraints—such as 20-second response limits and standardized 3-second vibration prompts—participants used their Apple Watches under typical conditions, including personalized vibration settings and occasional use of silence mode. The DOSE app also featured five distinct survey interfaces with two different questions, which may have contributed to longer response times by encouraging more deliberate reflection and requiring prolonged interactions to navigate a broader range of response options within each interface—unlike prior studies that used a single interface with one question and only 2–3 visible options, allowing for quick, repetitive one-tap responses. These findings provide encouraging evidence for the practical, real-world applicability of smartwatch-based ESM in middle-aged and older adults, expanding the reach and inclusivity of future research. Overall, these findings provide further evidence to challenge ageist beliefs that have contributed to a digital health divide for older adults [33].

Our findings also underscore the importance of user behavior as a key factor influencing ESM compliance. In this sample, some older adults who appeared to face challenges in consistently wearing or charging their devices. Irregular charging routines can lead to missed prompts due to power loss, preventing scheduled notifications from triggering and resulting in expired surveys. Additionally, tactile notifications can be easily missed during physical or cognitively demanding activities. While these factors present challenges, they also offer opportunities for future research to develop more user-specific and context-aware prompting strategies. Nevertheless, when devices were worn and prompts were detected, participants in our study demonstrated generally low response latency, with median device access times of 9 seconds for middle-aged adults and 9.5 seconds for older adults, These values compare favorably with smartphone-based ESM studies reporting median device access latencies exceeding 3 minutes [24, 34]. This low latency likely reflects the reduced friction of smartwatch access, greater tactile prompt salience, and a more lightweight interaction design. Importantly, however, these distributions exhibited a pronounced right-skewed upper tail, where a distinct portion of prompts (24.7% for middle-aged and 34.5% for older adults) exceeded an access latency of 1 minute. This pattern suggests that while immediate micro-interaction remains the norm, a meaningful subset of prompts are delayed by real-world context or ongoing tasks. Future studies built on the DOSE framework may further explore and characterize individuals’ temporal engagement patterns with ESM in relation to device use behaviors and contextual factors, with the goal of optimizing prompt delivery and further enhancing compliance.

A third contribution of the DOSE app is that it can be extended to deliver some ecological momentary interventions that require prompting. One such intervention is self-monitoring, a well-established behavior change technique that involves observing and recording one’s behavior [35]. This technique has a strong evidence base in the physical activity promotion literature [36]. Smartwatches have been used to prompt self-monitoring of physical activity recently [26]. A recent review further supports this approach of prompting use of behavior change techniques: brief, message-based prompting interventions can significantly improve both preventive health behaviors (e.g., smoking cessation) and clinical outcomes (e.g., diabetes management) [37]. The DOSE framework offers a foundation for new tools for delivering ecological momentary interventions that promote active engagement in healthcare and evidence-based behavior change strategies.

The DOSE app has several limitations. Perhaps most obviously, this app was written to work around limitations of the Apple Watch OS. It is possible that Apple Watch users differ systematically from users of other devices (e.g., Fitbit, Garmin, Google, or Samsung). The code will also not work on other operating systems so it would need to be paired with a companion app for other devices used in a study. Researchers sometimes provide study devices for participants to use during a study, the Apple Watch is not a low-budget device so this solution may not be possible for all researchers. Second, due to limited device-level contextual data, we were unable to distinguish whether prolonged access latencies reflected delayed intentional engagement or missed or non-detected notifications, and therefore could not fully disaggregate the observed heterogeneity in response timing. Accordingly, feasibility interpretations should only be understood primarily in relation to median latency and interface-level completion behavior. Third, the DOSE app was designed for ESM but some research questions require different sampling schemes (e.g., event-contingent recording). Likewise, although the app can deliver ecological momentary interventions on a random schedule, just-in-time adaptive interventions with tailoring variables and decision rules are not currently implemented. The current version of the the DOSE app also requires manual triggering of data uploads which limits just-in-time processing potential. The code would require more substantial modifications for other sampling schemes or to deliver just-in-time adaptive interventions. Finally, our feasibility evidence comes from a small, highly selective sample (78% White, 66% with a bachelor’s degree or higher, and 100% pre-existing Apple Watch owners) recruited from a broader physical activity study with eligibility criteria related to physical activity, mobility, pregnancy, and cognitive impairment. In addition, the older adult subgroup was predominantly female (7 women and 1 man), limiting our ability to assess whether feasibility outcomes differ by sex within this age group. Characteristics such as race/ethnicity, education, socioeconomic status, and other factors (e.g., digital literacy and access to premium hardware) likely influenced the high compliance rates and rapid response times observed in this study. Accordingly, these findings establish feasibility within this specific population and should not be interpreted as evidence of feasibility across the broader populations. Future validation should target larger, more diverse cohorts.

In sum, the DOSE app is an open-source tool created to work around limitations for ESM research on the Apple Watch. This app provides another option for researchers interested in sampling momentary states in users’ lives. Whereas most prior work with smartwatches has focused on young adults, this study established the feasibility of obtaining high-quality data from middle-aged and older adults.

## Materials and methods

### Ethics statement

The study was approved by the Pennsylvania State University Institutional Review Board (Approval Number: STUDY00023288) and conducted at the Pennsylvania State University. All participants provided informed consent prior to participation in the study.

We designed and programmed DOSE, an open-source iOS smartwatch template application, to address the aforementioned issues. A key innovation of the app is that it enables researchers to work around the limitation of 64 notifications on iOS by implementing a system that dynamically manages the notification schedule within the app. The DOSE template contains streamlined code that determines the number of notifications to send each day, the number of days into the future for which notifications should be scheduled, and the interval for automatic rescheduling of notifications. If three notifications are set to be sent per day and 20 days into the future are scheduled, the total scheduled notifications would be 60. If an interval of 5 days is set, the app will automatically reschedule the next 60 notifications every fifth day. A shorter interval is beneficial for participants traveling frequently across different time zones. This approach ensures a continuous flow of notifications for participants enrolled in ESM studies without reaching the 64-notification limit. These notifications can be delivered to participants’ Apple watch at evenly distributed random times, within pre-determined time windows. Researchers can also easily adjust the parameters within the code to increase or decrease the number of surveys to be delivered per day (equivalent to the number of time windows per day as only one notification is sent per window) or adjust the time intervals for each window by simply updating the corresponding values. This flexibility allows researchers to customize the notification (experience sampling) schedule according to their specific study requirements.

The DOSE framework was created using SwiftUI in Xcode. The back end of the current version of DOSE framework is based on Google Firebase Realtime Database as a data storage and a processing unit. With this framework, researchers have the flexibility to integrate various types of sensor data collection alongside ESM survey response data collection. As an illustrative example, the framework includes automatic collection of step counts and heart rate metric data through HealthKit during the predetermined study period. The full open-source code and conditions of use for this framework application are available for download via GitHub (https://github.com/iansulin/umich_dose). The workflow process for conducting an iOS smartwatch-based ESM study using the DOSE framework can be divided into three main stages: (1) compilation of the app to meet research design specifications, (2) participant onboarding and data collection, and (3) data processing for analysis.

### Step 1: App customization

**1.1.** **Working template**

Following the installation of the necessary software (Xcode and Simulators; see https://developer.apple.com/documentation/safari-developer-tools/installing-xcode-and-simulators), a new project using this framework can be initiated by importing the project folder of the source code files into Xcode. This will create a working template application that can be customized for specific research purposes. The template includes a collection of tools: (1) Utilities, (2) SurveyModules, and (3) Views. Detailed explanations for functions and parameters can be found in the comments throughout the code and in Chapter 2 (App Compilation) of the GitHub documentation.

Most study-specific customization can be completed by modifying existing template parameters and text fields rather than developing new application logic. In a typical deployment, researchers primarily configure survey questions, notification scheduling parameters, survey interface settings, and the Firebase Realtime Database connection. Based on informal observations during framework development and internal testing, researchers with basic familiarity with Xcode and iOS development workflows can typically complete these study-specific configurations in approximately one hour. The most common setup challenges involved Xcode provisioning, Apple Developer account configuration, and Firebase project setup rather than modification of the DOSE framework itself.

**1.2.** **Utilities**

The Utilities directory includes a range of essential components for the app, such as survey, storage, notification, HealthKit, and Firebase manager systems. Two crucial components that must be configured by individuals looking to utilize and integrate this framework app into a new, customized ESM app are the survey scheduling parameter definition and the database connection.

**Fig 4:**
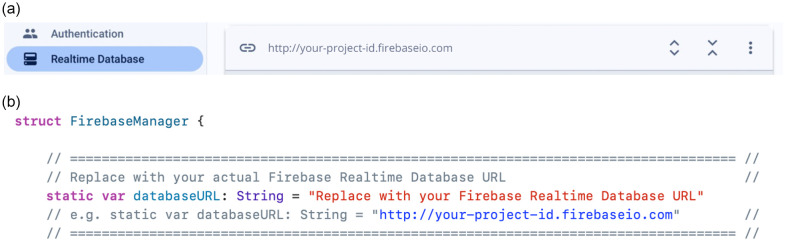
Database configuration.

**Database Configuration.** The DOSE framework leverages the Firebase REST API to interact with the Firebase Realtime Database. By adopting the Firebase REST API, the framework eliminates the need for the GoogleService-Info.plist file and SDK configuration, which can otherwise require substantial setup effort. When using the Firebase REST API, the only requirement is the Firebase Realtime Database URL. The URL typically follows the format “https://your-project-id.firebaseio.com” and can be found at the top of the Realtime Database section in the Firebase console (Fig 4a). Within the FirebaseManager, there are three specific functions where the URL needs to be configured: uploading notification details, uploading HealthKit data, and uploading survey response data. These instances are marked with a placeholder text “Replace with your Firebase Realtime Database URL” (Fig 4b). A comprehensive Security and Privacy Guide, including Firebase security rules and deployment best practices, is available in the DOSE GitHub repository: https://github.com/iansulin/UMich_DOSE/tree/main/Ch4_Security-and-Privacy.

**Fig 5:**
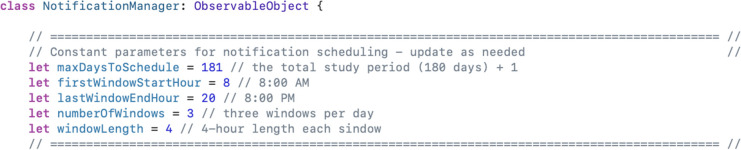
Survey scheduling.

**Survey Scheduling**. Survey scheduling requires the specification of five numeric constant parameters in the NotificationManager file: maxDayToSchedule, firstWindowStartHour, lastWindowEndHour, numberOfWindows and windowLength (Fig 5). The maxDayToSchedule parameter determines the entire study period during which participants will receive survey notifications. It represents the number of days plus one, as survey prompt delivery begins on the day following participants onboarding. For example, if the study period is 180 days, the value of this parameter should be set to 181. The firstWindowStartHour and the lastWindowEndHour parameters define the earliest and latest times for survey prompt delivery. The specified time should be in a 24-hour format, without AM/PM. For instance, if prompts are to be delivered between 8 AM and 8PM, the parameters should be set as 8 and 20, respectively, rather than 8 and 8. The numberOfWindows and the windowLength parameters determine the number of windows per day and the length of each window. Windows are currently constrained to be a constant size across the day. The product of these two parameters should correspond to the duration between the earliest and latest times for survey prompt delivery. For example, if the earliest time is 8 and the latest time is 20, resulting in a 12-hour duration, and the length of each window is 4 hours, then the number of windows should be 3 to achieve a total duration of 12 hours. Depending on the specific requirements and features of the app, additional parameters may be added to customize the scheduling system.

**1.3. Survey Module** **s**

**Fig 6:**
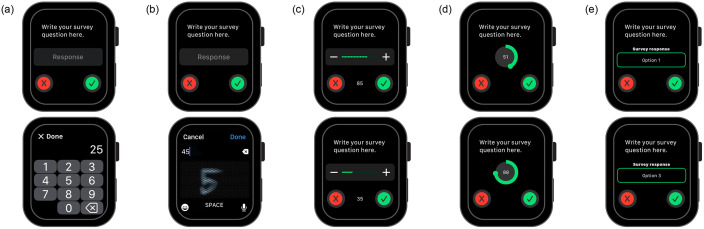
Input interfaces for the survey module: (a) Numeric Keypad, (b) Scribble/Dictation, (c) Step Slider, (d) Circular Slider, and (e) Scrollable List.

**Survey Interface.** The SurveyModule directory comprises five survey interfaces (rand_survey_1–5), each featuring a distinct design with different layouts. Among them, two interfaces facilitate open-ended responses using user input. One interface incorporates a digit pad for numeric responses (rand_survey_1; Fig 6a), while the other provides users with choices such as the regular Apple Watch keypad, scribbles, or voice typing for character or numeric input (rand_survey_2; Fig 6b). Additionally, two interfaces offer slider scales. One employs a stepper slider, which allows individuals to increment or decrement a value using a two-segment control (rand_survey_3; Fig 6c). The other employs a circular slider (rand_survey_4; Fig 6d). The slider scale interfaces can be controlled through hand gestures (swiping or tapping) or by turning the Digital Crown, the rounded knurled knob on the side of the Apple Watch. Lastly, one interface contains a picker for multiple-choice questionnaires, that displays one or more scrollable lists of distinct values that users can choose from (rand_survey_5; Fig 6e). Each survey interface includes two buttons at the bottom of the screen. The red button on the left enables users cancel the survey without submitting their response, while the green button on the right side of the screen allows users to submit their response.

**Fig 7:**
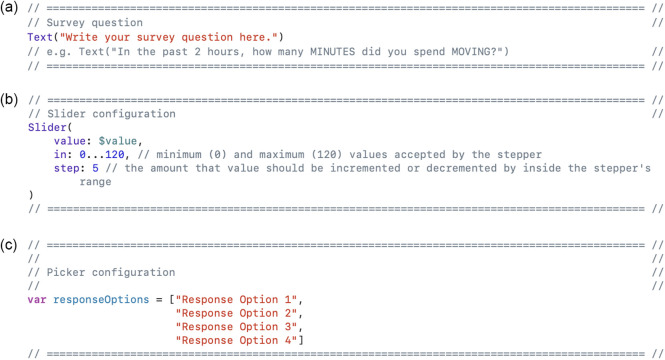
Survey question (a), slider configuration (b) and picker configuration (c).

**Survey Design.** The survey question statements can be easily updated by replacing the placeholder text “Write your survey question here” under the commented line “// Survey question” in each survey module (Fig 7a). For slider scales, the parameters for the minimum value, maximum value, and increment (for stepper slider) of the response can be modified under the commented line “// Slider configuration” (Fig 7b). The picker interface lists can be updated by replacing the choice options under the commented line “// Picker configuration” (Fig 7c). Each of the five survey modules is numbered from 1 to 5. When a user submits their responses, the survey response is sent to the survey database along with the corresponding survey module number, dates and times of survey delivery, opening, and submission. In cases where the survey was dismissed, a numeric value of 99999 is sent to the database as the survey response. If a participant opens a survey but submits it without entering any responses, the submission is still recorded, but the response field is left blank.

**1.4.** **Views**

**Fig 8:**
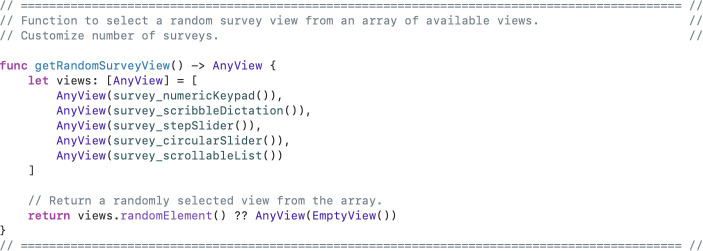
Survey selection.

**Survey Selection**. The Views directory encompasses the entire app interface and crucial mechanics of the app. Within this directory, there are various views available, and one of them is the MainView. The MainView includes a function called getRandomSurveyView (Fig 8) that allows for the selection of specific survey modules for future survey deliveries. By default, all five modules are included in the function, but it can be easily updated by keeping or removing lines that include the specific name of the desired survey module. The surveys are randomly chosen and delivered at random times within pre-defined time windows throughout the specified study period.

**Fig 9:**
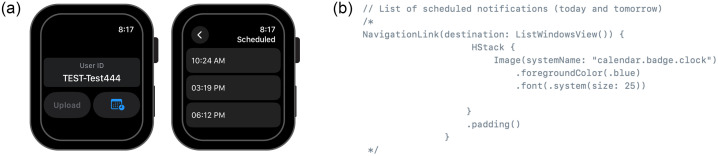
Survey schedule display on the app (a) and block-commented code for schedule display in the MainView (b).

**Survey Schedule Display.** The MainView also defines the layout of the app’s main screen. The DOSE framework app’s main screen comprises three primary elements: the User ID display, an upload button for uploading HealthKit data, and a calendar icon button for future survey notification schedule display (Fig 9a). While hiding the survey delivery time is a standard practice in ESM studies to minimize bias and maintain ecological validity, including a survey delivery schedule display button in prototype ESM apps can be valuable for testing purposes. It facilitates debugging, customization, and experimentation with survey timing. However, once testing is complete, it is recommended to remove the display button from the actual ESM survey app before deploying it. To remove the display button, we can use a block comment method. Typically this involves surrounding the relevant code section with block comment symbols, such as/* and */, effectively commenting out the code. This way, the code will be temporarily disabled while preserving a backup of the original code. The responsible code chunk is located under the commented line “// List of scheduled notifications (today and tomorrow)” (Fig 9b).

**App Deployment**: Finally, by using the “Product – Archive” feature in Xcode, a new app for a native, ESM watch app can be created from the source code.

### Step 2. Participant onboarding and data collection

**2.1. Push** **Notification Permission**

**Fig 10:**
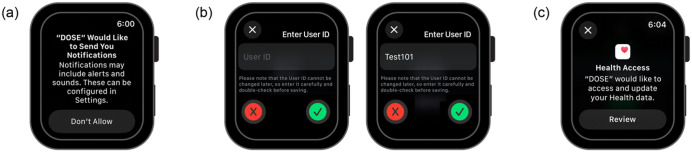
Notification permission grant (a), user ID selection (b), and HealthKit data access authorization (c).

After downloading the app from App Store or TestFlight, study participants are immediately prompted to grant notification permission (Fig 10a). This permission is necessary as the app relies on push notifications to deliver EMS surveys to the participants.

**2.2 U** **ser ID Selection**

Once notification permission is granted, study participants proceed to register with a User ID by typing it into a designated input box displayed on the top of the app’s home screen (Fig 10b). The User ID can either be chosen by the participant themselves or assigned by the researchers. It must consist of both alphabetical letters and numeric values. This User ID is used as a pseudonymous identifier to link participant data collected through the app within the Firebase Realtime Database (note that it is not used for authentication or cryptographic encryption). Upon initial registration, the User ID is associated with a device-specific identifier generated by the operating system, enabling linkage of HealthKit data collected from that specific Apple Watch during the study period. Accordingly, the User ID is fixed for the duration of the study and cannot be changed unless the participant transitions to a new device. All data streams are linked to the User ID and stored in the Firebase Realtime Database. If a study involves redistribution of devices across participants, the device must be reset prior to reassignment to ensure separation of data across users and prevent cross-participant data linkage.

**2.2. Auth** **orizing Access to HealthKit Data**

HealthKit serves as a central repository for health and fitness data on iOS. Apple provides an API that enables developers to request various types of continuous data, including step counts, heart rates, calories burned, and sleep patterns. Just like notification permission, when integrating HealthKit capability into an app, the app needs to request permission from the user to access and retrieve data from HealthKit, as well as to transfer it to a database. These permissions can be obtained by tapping the “Upload” button located at the bottom left of the app’s home screen (Fig 10c**)**. Once the permissions are granted, the app can periodically retrieve data from HealthKit. In this framework, specifically, the step count and heart rate data are retrieved and transferred to Firebase Realtime Database with the User ID whenever the “Upload” button is tapped. Upon tapping, the data collected between the previous tap (or the last upload point) and the current tap (the present time) is fetched and transferred. This retrieval must be initiated manually by the user by pressing the “Upload” button. While automatic background retrieval, with a researcher-defined period, is planned for future versions, it has not been implemented in the current version of the framework.

### Step 3. Data processing

**3.1. F** **irebase directory structure**

**Fig 11:**
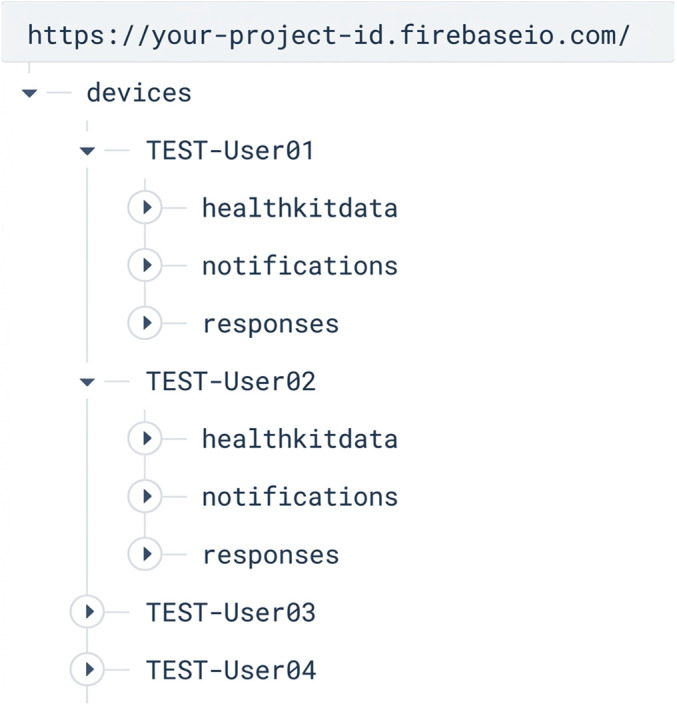
Firebase directory structure with parent and child nodes.

With the DOSE framework app, all survey responses and HealthKit data are transmitted to Google Firebase Realtime Database in real-time as users submit or upload them. In Firebase Realtime Database, data is organized in a tree-like structure of nodes and keys. A node acts like a folder and can contain values or other nested nodes, while a key is a label identifying each piece of data. In the DOSE app, all data is organized under a single parent node called “devices,” which contains child nodes named after each participants’ User ID. Each of these child nodes has three additional child nodes: “healthkitdata”, “notifications” and “responses” (Fig 11). These nodes are automatically created when a study participant opens their survey notifications or upload HealthKit data for the first time. The current implementation does not use Firebase Authentication; instead, data access is controlled through Firebase Realtime Database security rules configured by the researcher at deployment, and all data transmission occurs over HTTPS/TLS via the Firebase REST API.

**3.2. Survey Response and Notification Data**

**Fig 12:**
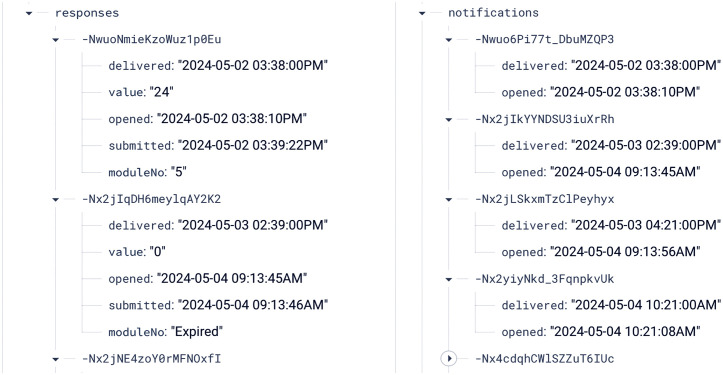
Survey response and notification data structure in Firebase with parent nodes, child nodes, and keys.

The response data for the ESM survey is stored in two nodes in Firebase Realtime Database: “responses” and “notifications” (Fig 12). The “responses” node contains keys for surveys that have been submitted or dismissed, including date and time information for when the survey was delivered, opened, submitted (or dismissed), along with the response value and survey module number. If a previous survey has not been completed and a new survey is delivered, the previous survey is marked as “expired” when the new survey is sent. Even if a response is later submitted for the previous survey in this scenario, it remains marked as “expired” in the survey module number field. The “notifications” node stores keys for surveys that were delivered and opened but never submitted or dismissed. It contains the date and time information for when the notification was delivered and opened. The purpose of the “notifications” node is to facilitate additional investigation into participants’ non-compliance behaviors.

As of June 2025, the Apple Watch does not provide a direct way to track or retrieve notification delivery status or timestamps for notifications without user interactions, such as opening the notification. For instance, if a user swipes the notification to remove it, it is not possible to track the notification delivery status. The DOSE framework relies on the UNUserNotifications API to locally schedule notifications. Because notifications are stored directly on the device once scheduled, their delivery does not depend on network connectivity, which is the primary known cause of notification loss [38]. Under normal conditions, this approach ensures virtually 100% reliability—a locally scheduled notification might not appear only if user has disabled notifications for the DOSE app or if the device is in a Focus mode (such as do not disturb, sleep, or work) [39].

**3.3.** **HealthKit Data**

**Fig 13:**
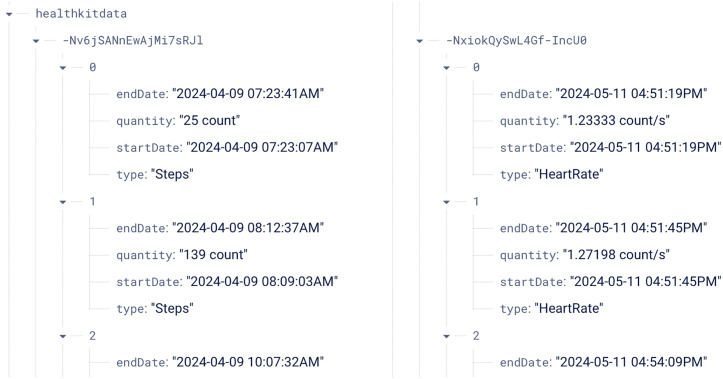
HealthKit data structure in Firebase.

HealthKit records data as events of variable durations with discrete start and end times. The “healthkitdata” node consists of four keys: “startDateTime,” “endDateTime,” “quantity,” and “type.” Within these files, step counts and heart rates from HealthKit data are stored as raw, non-aggregated information (Fig 13). They are represented using predefined structures that include start date/time, end date/time, type (e.g., step counts, heart rates) and quantity. In HealthKit, the time intervals at which raw step counts and heart rates are measured can vary depending on the device. Different devices have varying capabilities and settings for capturing these data, with intervals ranging from a few seconds to a longer duration in 10–15 minutes. The startDateTime and endDateTime record the interval within which a participant’s device captures the data. The unit of measurement associated with the quantity is based on the participant’s device settings, allowing for potential variations in the units associated with HealthKit data according to user preferences. The quantity within these data structures represents the measure value between the startDateTime and endDateTime (e.g., 100 steps [2024/02/03 11:23 AM – 2024/02/03 11:34 PM], 1.6 beats per second [2024/02/03 1:41 PM – 2024/02/03 1:44 PM).

**3.4.** **File Conversion From JSON to CSV**

In Firebase Realtime Database, data is organized in a hierarchical tree structure, and each node within the tree can store data in JSON format. To download the JSON data from the desired location in the database tree, you can access the Firebase Realtime Database console and click on the “Export JSON” button, typically found at the top-right corner of the data view panel. This initiates the download of a file called “database-export.json” containing the selected data. It is worth noting that while the JSON format allows for flexible organization and representation of data within the console, it may not be optimal for certain types of analyses. To address this, we have developed a Python script specifically for researchers to download, process, and convert the data to a flat CSV file for analysis. The script, along with detailed instructions, can be downloaded via GitHub (https://github.com/iansulin/umich_dose).

### Feasibility study

#### Objectives.

We conducted a feasibility study to determine if it is possible to obtain valid data from Apple Watch-based ESM data collection using the new DOSE app. This app delivered random survey notifications (3 times daily) over a 28-day experience sampling period (84 prompts total) that exceeded the current iOS limit on scheduling notifications. Secondary objectives included assessing overall survey completion rates and evaluating device access time and interface usage time during micro-interactions. Micro-interactions are typically understood to be surveys that can be completed within 4 seconds or less [9,25]. Access time refers to the duration from when a survey prompt is delivered to when the survey is opened, while interface usage time refers to the duration from when the survey is opened to when it is submitted.

### Recruitment and participants

We recruited adults (aged 40–80 years) who owned an iPhone and an Apple Watch with iOS v16 (or higher) and watchOS version 8.1 (or higher) through online social media platforms between January and October 2024 (N = 20). Participants were excluded if they reported 90 minutes or more of moderate-vigorous intensity physical activity in the past week, any contraindications to physical activity on the Physical Activity Readiness Questionnaire for Everyone (PAR-Q + ; [40]), had any mobility restrictions that interfered with unassisted physical activity, were pregnant or planning to become pregnant during the study, were concurrently participating in another study involving physical activity or weight loss, planned to have surgery or relocate during the study, or had been diagnosed with mild cognitive impairment or Alzheimer’s disease or dementia. Two participants withdrew within 24 hours without providing a specific reason. As a result, data from the remaining 18 participants were used in the analysis (Table 3). Participants were compensated up to $250 for completing assessments (including activity monitoring and cognitive function assessments not reported here) but none of the payments were contingent on using or responding to prompts from the DOSE app.

**Table 3:** Demographic characteristics of sample that completed the study (N = 18).

	Total	Middle-aged adults	Older adults
**All**	18 (100%)	10 (56%)	8 (44%)
**Sex**			
Male	7 (39%)	6 (60%)	1 (13%)
Female	11 (61%)	4 (40%)	7 (87%)
**Age** (in years)	55.3 (9.2)	48.6 (6.2)	63.3 (1.6)
**Ethnicity**			
Hispanic/Latino	1 (6%)	1 (10%)	0
Non-Hispanic/Latino	17 (94%)	9 (90%)	8 (100%)
**Race**			
White	14 (78%)	7 (70%)	7 (87%)
Black/African American	2 (11%)	1 (10%)	1 (13%)
Asian	2 (11%)	2 (20%)	0
**Education**			
Some college (no degree)	2 (11%)	2 (20%)	0
Associate’s degree	2 (11%)	0	2 (25%)
Bachelor’s degree	8 (44%)	4 (40%)	4 (50%)
Master’s degree	1 (6%)	1 (10%)	0
Doctoral degree	4 (22%)	3 (30%)	1 (13%)
Refuse to answer	1 (6%)	0	1 (13%)

### Survey questions and scheduling

For experience sampling, two survey questions were created: (1) “In the past 2 hours, how many minutes did you spend *exercising*?” and (2) “In the past 2 hours, how many minutes did you spend *moving*?” Each of these questions was paired with the five survey interfaces introduced in the 1.3 SurveyModule section, resulting in a total of ten surveys in the SurveyModule. The scrollable list interface offered options ranging from 0 to 120 minutes in 5-minute intervals. The step slider interface allowed users to increase or decrease values within the same range and intervals using + or – buttons. The circular slider interface also provided options from 0 to 120 minutes, with increments or decrements of 1 minute. All interfaces defaulted to 0. During the 28-day study period, notification prompts were delivered three times per day over a 12-hour period, between 8 AM and 8 PM. These prompts were randomly distributed across three 4-hour windows throughout the day. Among the ten possible survey configurations (= 2 questions X 5 interfaces), only one was randomly selected and delivered at a time. Notifications were scheduled for ten days, with three notifications each day, totaling 30 notifications at once. Every 3 days, a new set of 30 notifications was automatically rescheduled.

### Measures

Survey completion rates for the experience sampling were calculated as a proportion of the number of surveys submitted out of the number of surveys scheduled. To capture behaviors in real-world settings, participants were not instructed to keep their devices unusually charged or wear them more frequently than usual; instead, they were asked to maintain their regular Apple Watch usage. As a result, some participants occasionally forgot to charge their watches or wear them on certain days. To account for periods when the watch was not worn, watch non-wear windows were identified using heart rate data retrieved from HealthKit at the end of the study. Specifically, for each 4-hour survey deployment window, a window was classified as non-wear if heart rate data were unavailable for at least 1 hour of that 4-hour period (e.g., if a device samples every 15 minutes, at least 4 observations are required to be considered a valid wear window). To our knowledge, there is currently no validated method for defining Apple Watch non-wear time. Therefore, we adopted a pragmatic coverage-based approach that could be applied consistently across participants while accounting for variability in heart rate sampling frequency across Apple Watch models and usage conditions. Because a 4-hour block was considered valid as long as it met this minimum 1-hour threshold, prompts deployed during any remaining transient non-wear hours within that block were retained in the analysis and counted as missed; thus, this approach provides a conservative lower-bound that may slightly deflate true completion rate estimates. The proportion of non-wear windows was calculated for each participant, and survey observations corresponding to non-wear windows were excluded from analyses. Micro-interaction rates were calculated as the percentage of interface usage lasting 4 seconds or less.

### Open practices

**Code availability**: The source code for the DOSE platform developed by I.K. and S.K. is available for public access. It can be found on GitHub (https://github.com/iansulin/umich_dose) and is provided under an open-source license. Users are encouraged to explore, contribute, and provide feedback.
